# Examining the Relationship Between Hospital Nurses' Structural Empowerment, Missed Nursing Care and Quality of Care: A Cross‐Sectional Study

**DOI:** 10.1111/jocn.17816

**Published:** 2025-05-23

**Authors:** Arlene Travis, Joyce J. Fitzpatrick

**Affiliations:** ^1^ Frances Payne Bolton School of Nursing Case Western Reserve University Cleveland Ohio USA; ^2^ University of Mount Saint Vincent Bronx New York USA; ^3^ Marian K. Shaughnessy Nurse Leadership Academy, Frances Payne Bolton School of Nursing Case Western Reserve University Cleveland Ohio USA

## Abstract

**Aim:**

To examine relationships between structural empowerment, missed nursing care and quality of care among hospital‐based, direct‐care nurses.

**Design:**

Cross‐sectional study.

**Methods:**

A convenience sample of 161 nurses completed the Conditions for Work Effectiveness‐II Questionnaire, the MISSCARE and a single‐question rating of quality of care. Correlation, *T*‐tests, regression and ANOVA were used to analyse data.

**Results:**

Nurses reported high structural empowerment (total CWEQ‐II = 22.8). Higher empowerment was significantly correlated with less missed care. Most nurses (77.7%) worked at Magnet hospitals; however, no difference in missed care was found between Magnet and non‐Magnet nurses. The average number of patients on the last shift was 5.1. The number of patients cared for was not significantly correlated to missed care; however, nurses' perceptions of better staffing adequacy, teamwork and job satisfaction were. Nurses who intended to leave (25.5%) missed more care. Intention to leave and access to resources predicted missed care.

**Conclusion:**

This appears to be the first study examining the relationship between structural empowerment and missed care, demonstrating that higher empowerment was related to greater nurse work effectiveness and improved care delivery. Work environment factors, specifically subjective perceptions of staffing and resource adequacy, were linked to missed care, while nurse–patient ratio was not. Subjective factors may contribute more to missed care than is recognised.

**Implications:**

Creating and sustaining empowering work environments, ensuring resource adequacy and enhancing factors that promote retention may reduce missed care. No patient/public contribution.

**Financial Conflicts of Interest:**

None.

**Reporting Method:**

STROBE checklist for cross‐sectional studies.

**Summary**

- Higher clinical nurse empowerment was linked to improved care delivery and less missed care.
- Increasing empowerment and visibility may reduce missed care.
- Subjective perceptions of staffing adequacy were correlated to missed care, while nurse‐patient ratio was not.
- More research is needed on how subjective factors and nurses internal processes may be related to missed care.

## 1 Introduction

Missed nursing care (MNC), was defined by Kalisch et al. (2009) as ‘any aspect of required patient care that is omitted (either in part or in whole) or delayed’. It has been extensively studied, primarily in acute care settings, over the last two decades, and found to occur regularly and frequently in hospitals in the US and internationally (Ball et al. 2018; Gong et al. 2025; Lee and Kalisch 2021). Most (55%–98%) of nurses report missing at least one nursing care activity recently (Griffiths et al. 2018) with some items, such as ambulation, turning and mouth care reported missed > 75% of the time. Studies have consistently linked MNC to negative patient outcomes, such as increased mortality and in‐hospital adverse events (Ball et al. 2018; Recio‐Saucedo et al. 2018). Missed care has also been associated with negative consequences for nurses, including lower nurse job satisfaction, increased burnout, higher intention to leave and moral distress (Stemmer et al. 2022; Xu et al. 2024). The economic impact of MNC on healthcare organisations has not yet been directly examined; however, in the current context of reimbursement models which link payment to performance on quality benchmarks (many of which are nurse sensitive), it is likely that MNC negatively impacts hospital finances. Substantial research has been conducted on the topic and numerous causes and contributors have been identified, yet missed care persists as common and prevalent problem, suggesting that daily, hospitalised patients around the world continue to not receive all the care they require.

## 2 Background

MNC (also studied as unfinished care, care left undone and rationed care) (Jones et al. 2015) is conceptualised as occurring when demands for patient care exceed available patient care resources, requiring nurses to prioritise care activities. Kalisch et al. (2009) use a structure‐process‐outcomes framework to describe the phenomenon of MNC. The Missed Care Model (Kalisch et al. 2009) describes MNC as occurring when antecedents within the work environment interact with nurses internal processes (values, experience, norms) and the nursing process to necessitate decisions about what care will be delivered and what will be curtailed in situations in which patient care demands exceed available resources. Studies have identified multiple antecedents, primarily at the organisational level. Issues surrounding staffing, the quality of the work environment, and communication and teamwork have been identified as the most frequent reasons for missed care (Chiappinotto et al. 2022; Kalisch and Xie 2014). Improvements in both staffing and work environments have been associated with a reduction in MNC (Lake et al. 2020).

Despite these findings, very few interventions to reduce missed care have been developed or implemented (Schubert et al. 2021). It is likely that additional important antecedents to missed care remain unidentified. This is evidenced by the findings of a state of the science review by Jones et al. (2015), in which factors identified as antecedents of missed care across the 54 studies included in the review were entered into various regression models, and the models only accounted for 12%–32% of the variance in missed care. Researchers have recommended that more research be done on underexamined factors in the work environment and on individual aspects of the practice environment that may contribute to MNC (Zhao et al. 2020).

Much emphasis has been placed in recent years on the importance of positive nurse practice environments for supporting optimal nurse and patient outcomes (Mabona et al. 2022) and on understanding the characteristics of these environments. Structural empowerment (SE) is frequently cited as a key element of positive practice environments, evidenced by its designation as one of four core components of the Magnet work environment.

SE is described as an employee's perception of their access to structures within the workplace which facilitate the completion of work (Kanter 1993; Laschinger 2012). The concept of SE as applied to nursing research is derived from Kanter's (1993) theory of workplace empowerment and the structural determinants of organisational behaviour. Kanter proposes that an employee's performance in the workplace is significantly affected by their access to empowerment structures in the workplace, specifically the structures of opportunity, information, support and resources. Access to empowerment structures significantly influences the employee's performance in the workplace, potentially even more so than individual characteristics. Employees who are able to gain access to these structures are supported in accomplishing their work and are more likely to advance within organisations. Power is required to access the structures. Two types of power are described: formal and informal power. Formal power derives from job activities and their perceived importance to organisational goals (which provide visibility) and is characterised by flexibility and independence in decision‐making. Informal power derives from relationships and alliances with people at all levels of the organisation. According to Kanter, empowerment provides a person with agency, that is, the ability to get things done in the organisation.

Laschinger et al. (2001) expanded upon Kanter's theory, incorporating the concept of psychological empowerment into the theory, proposing that SE leads to employee psychological empowerment, which in turn leads to positive organisational behaviours and feelings. These include increased work effectiveness, higher job satisfaction and a sense of meaning in work. For clinical nurses, whose work is the delivery of patient care, getting things done would manifest as completing care delivery, that is, in missing less care. A sense of meaning in work would include the perception that the care they deliver is of a high quality.

SE and its relationship to patient and nurse outcomes has been widely studied. Among nurses, it has been linked to better nurse job performance (Ta'an et al. 2020); greater nurse organisational commitment, job satisfaction and psychological empowerment (Fragkos et al. 2020); greater work engagement (García‐Sierra and Fernández‐Castro 2018); lower intention to leave (Arslan Yürümezoğlu and Kocaman 2019); and lower burnout (Şenol Çelik et al. 2024). It has also been linked to higher nurse‐assessed quality of care and improved safety culture (Goedhart et al. 2017).

SE is theorised to lead to work effectiveness and a sense of meaning in work (Laschinger et al. 2001). Since providing patient care is the primary work of nurses, MNC can be thought of as an indicator of work effectiveness, i.e., the more care missed, the lower the work effectiveness; and conversely, the less care missed, the greater the work effectiveness. The relationship between SE and MNC has not yet been examined. The primary aims of this study were to examine the relationship between SE and MNC, and to examine the relationship between MNC and nurses perceptions of quality of care. The primary research questions were:

1. What is the relationship between the SE of clinical nurses and nurse‐reported MNC?
2. What is the relationship between MNC and nurses' perception of quality of patient care (QOC)?

Exploratory analyses of other data collected were also deemed possible.

## 3 Methods

### 3.1 Design

A descriptive, correlational, cross‐sectional design was employed to examine the relationships between SE, MNC and nurse‐assessed quality of care among direct‐care, clinical nurses. The Strengthening of Reporting of Observational Studies in Epidemiology (STROBE) guidelines and checklist for cross‐sectional studies were followed (von Elm et al. 2007) (File S1).

### 3.2 Sample and Setting

A convenience sample of hospital‐based clinical nurses was recruited from nurses attending the 2018 American Nurses Credentialing Center (ANCC) National Magnet conference (October 24–27, 2018). Registered nurses working 30 or more hours per week, delivering direct patient care on adult medical–surgical units of acute care hospitals, were eligible to participate. Nurses working in areas other than acute care medical–surgical units, with populations other than adults, or who did not deliver direct care as their primary role (such as managers, educators, other specialty nurses) were ineligible.

### 3.3 Variables and Measures

The primary variables examined in the study were SE, MNC and nurse‐assessed quality of patient care. SE was measured with the Conditions for Work Effectiveness Questionnaire‐II (CWEQ‐II) (Laschinger 2012), a 19‐item questionnaire divided into six subscales. The subscales measure nurses perceptions of their access to the workplace empowerment structures of opportunity, information, resources and support, and perceptions of their formal and informal power to access the structures. Responses range from 1 to 5 (low to high). An average score for each subscale is calculated. These are aggregated to produce a total SE score (CWEQ‐II total), with a range of 6–30. Scores of 6–13 represent low empowerment, 14–22 indicate moderate empowerment and 23–30 indicate high empowerment. The CWEQ‐II has been widely used in nursing research. Laschinger (2012) reports Cronbach's alpha for the scale and its subscales as ranging from 0.67 to 0.89.

MNC was measured with the MISSCARE survey (Kalisch and Williams 2009). The survey has three parts: a Demographics section; Part A (nursing care items missed), and Part B (reasons for missed care). Part A elicits nurses' perceptions of the frequency at which 24 specific nursing care items are missed, with response options of 0 (never), 1 (rarely) 2 (occasionally), 3 (frequently) and 4 (always). Part B elicits nurses' perceptions of the relative significance of 17 specific reasons for MNC, with response options of 0 (not a reason), 1 (minor reason), 2 (moderate reason) and 3 (significant reason). The 17 reasons are grouped into 3 subscales (categories), which are: (1) problems with labour resources (5 questions); (2) problems with material resources (3 questions) and (3) problems with communication and teamwork (9 questions).

Psychometric properties of the MISSCARE were reported by Kalisch and Williams (2009). The questionnaire demonstrated a content validity index = 0.89. Reliability (test–retest) coefficients for Part A (missed care items) were reported as 0.87. Reliability coefficients for Part B (reasons for missed care) were reported as 0.86. Cronbach's alpha for part B and its subscales ranged from 0.64–0.86. Cronbach's alpha coefficients for Part A were not calculated, as the authors deemed the 24 nursing care items on part A to be unrelated to one another, and their completion not contingent upon completing the other items (Kalisch and Williams 2009).

Quality of care was measured using a single‐question measure: ‘How would you describe the quality of nursing care delivered to patients in your unit?’ Response options are 1 (poor), 2 (fair), 3 (good) and 4 (excellent). McHugh and Stimpfel (2012) confirmed the accuracy this question as a reflection of quality of hospital care through a secondary analysis of a large dataset collected from 396 hospitals. The analysis demonstrated that nurses perceptions of quality of care were significantly correlated with outcomes of care, care processes and patient satisfaction scores. The question is used frequently in studies to which involve assessment of quality of care.

The Demographics portion of the MISSCARE collects information on nurse characteristics, hospital characteristics, patients cared for on the last shift; job, role and teamwork satisfaction, intention to leave and staffing adequacy. Nurses rate their job satisfaction, role satisfaction and teamwork satisfaction on a 5‐point Likert scale ranging from 1 (very dissatisfied) to 5 (very satisfied). Nurses report how often they perceive staffing on their unit to be adequate as 0%, 25%, 50%, 75%, or 100% of the time. For intention to leave, response options are yes, no, within 6 months, or within 1 year. Intention to leave was dichotomised to yes/no for data analysis.

### 3.4 Sample Size Calculation

A sample size calculation was performed using G* Power, with parameters of (*α*) = 0.05; power = 0.80, effect size = 0.3. The calculation indicated that a sample size of 84 was required for a two‐tailed hypothesis.

### 3.5 Human Subjects Protection

The study was determined minimal risk and exempt from review by Case Western Reserve University's Institutional Review Board.

### 3.6 Procedures

The investigator and a research assistant attended the 2018 American Nurses Credentialing Center Magnet conference which took place 24 October to 27 October, 2018 in Denver, Colorado. The conference was attended by 10,465 nurses (American Nurses Credentialing Center 2019). Data were collected using paper and pen questionnaires. The investigator and the assistant were seated at a table in the exhibition hall. Nurses visiting the exhibition hall could approach the table if they wished to and volunteer to participate. Nurses who completed the questionnaires received a $5 Starbucks gift card.

### 3.7 Data Analysis

Data were analysed using IBM SPSS Statistics, Version 28 (Armonk, NY, USA). Descriptive statistics were calculated to summarise demographic characteristics. Frequency distributions were generated for categorical data, while mean, median, mode and standard deviation were calculated for non‐categorical variables. Mean, median and standard deviation were calculated for the CWEQ‐II total score and each subscale. Frequency distributions were generated for responses to MISSCARE Part A (individual nursing activities missed) and Part B (reasons for missed care). Means and SD for all 24 items on Part A were calculated, and a total missed care score was derived by averaging the means of each of the 24 questions on Part A. Similarly, for MISSCARE Part B, means and SD for part B were computed, and total scores were calculated for each of its three subscales.

Normality for continuous variables was evaluated using the Shapiro–Wilk test to determine the appropriate statistical approach for analyses. Spearman correlation was used to assess associations between primary variables. Linear regression and ANOVA were conducted as exploratory analyses to examine relationships between key predictors of missed care and to compare levels of missed care across groups. A *p‐*value of < 0.05 was used to indicate statistical significance.

Missing data were minimal, and no imputation was performed. Analyses were conducted using pairwise deletion, allowing all available data to be used in each statistical test while preserving the integrity of the dataset.

## 4 Results

### 4.1 Sample Characteristics

A total of 185 nurses were enrolled and completed study surveys. Twenty‐four nurses did not meet inclusion and exclusion criteria. These included those who did not primarily deliver direct care, managers and nurses who did not work on adult medical–surgical units, leaving a final sample of 161. See Figure S1 for the study flow chart. Table 1 presents the sample characteristics.

**TABLE 1:** Sample characteristics (*n* = 161).

Variable	*N*	%
Licence		
RN	156	96.9
LPN	4	2.5
Unknown	1	0.6
Gender		
Female	146	90.7
Male	14	8.7
Unknown	1	0.6
Highest educational level		
ADN (Associate Degree)	22	13.7
BSN	117	72.7
Master's	20	12.4
Doctoral	1	0.6
Unknown	1	0.6
Age		
< 25	16	9.9
25–34	68	42.2
35–44	38	23.6
45–54	26	16.1
55–64	11	6.8
> 65	1	0.6
Unknown	1	0.6
Unit type		
Medical	30	18.6
Surgical	17	10.6
Mixed medical and surgical	113	70.2
Unknown	1	0.6
Experience as a nurse		
< 6 Months	1	0.6
6 Months—2 years	22	13.7
> 2–5 years	58	36.0
> 5–10 Years	32	19.9
> 10–20 Years	31	19.3
> 20 Years	16	9.9
Missing	1	0.6
Magnet hospital		
Yes	125	77.6
No	36	22.4
Region of US (US census regions)		
Northeast	45	28
Midwest	35	21.7
South	36	22.4
West	45	28

The majority of the participants were RNs (96.9%), female (90.7%), held a bachelor of science (BSN) degree (72.7%), were from Magnet hospitals (77.6%), and worked on mixed medical–surgical units (70.2%). Over half (52.2%) of the participants were under the age of 35, and 50% had ≤ 5 years of experience as a nurse. Participants were from all four geographic regions of the US (Northeast, South, Midwest and West.).

Table 2 summarises nurses' job and role satisfaction, satisfaction with teamwork on the unit, perceptions of staffing adequacy and intention to leave. Most participants (85.1%) reported being satisfied or very satisfied with their jobs, and only 4% reported being dissatisfied or very dissatisfied. The majority (95.7%) were satisfied or very satisfied with their role as a nurse: 61.5% reported being very satisfied with their role. No nurses reported being very dissatisfied with their role. Satisfaction with teamwork was also high, with 83.8% being either satisfied or very satisfied with the level of teamwork on their unit. Twenty‐five percent (25.5%) reported intending to leave their position. Regarding staffing adequacy, 52.2% of participants felt that staffing on their unit was adequate 75% of the time, and 29.8% reported it being adequate only 50% of the time. The mean number of patients cared for on the last shift was 5.1 (SD = 1.23) (see Table 5).

**TABLE 2:** Additional nurse characteristics.

Variable	*N*	%
Job satisfaction		
Very dissatisfied	1	0.6
Dissatisfied	5	3.1
Neutral	18	11.2
Satisfied	91	56.5
Very satisfied	46	28.6
Satisfaction with role		
Very dissatisfied	0	0
Dissatisfied	1	0.6
Neutral	6	3.7
Satisfied	55	34.2
Very satisfied	99	61.5
Satisfaction with teamwork		
Very dissatisfied	1	0.6
Dissatisfied	7	4.3
Neutral	18	11.2
Satisfied	78	48.4
Very satisfied	57	35.4
Intend to leave position		
Yes	41	25.5
No	120	74.5
Staffing adequacy (percent of time staffing is adequate)		
0%	1	0.6
25%	17	10.6
50%	48	29.8
75%	84	52.2
100%	11	6.8
Intention to leave		
Yes	41	25.5
No	120	75.5

### 4.2 Structural Empowerment (CWEQ‐II)

Nurses reported a high level of perceived SE, with a mean CWEQ‐II score of 22.80 (SD 3.39). Among the empowerment subscales, the highest scores were reported for access to opportunity (M = 4.17, SD = 0.61), which reflects opportunities for learning, growth and advancement. The lowest scores were for access to resources in the workplace (M = 3.27, SD = 0.90). Of the two types of power measured, nurses reported higher informal power (M = 4.08, SD = 0.65) than formal power (M = 3.50, SD = 0.85). Formal power is derived from job activities and their perceived importance to organisational goals, and the employee's visibility within the organisation. Informal power derives from relationships and alliances at all levels of the organisation. All of the CWE‐II subscales demonstrated good internal consistency. Cronbach's alpha ranged from 0.75 to 0.82, indicating acceptable to strong reliability (Table 3).

**TABLE 3:** Structural empowerment total score and subscales (*n* = 161).

Variable	Mean	SD	Cronbach *α*
Total SE (CWEQ‐II total score)	22.80	3.38	0.81
Opportunity	4.17	0.61	0.82
Information	3.94	0.85	0.77
Support	3.81	0.83	0.75
Resources	3.27	0.90	0.77
Formal power	3.53	0.85	0.76
Informal power	4.08	0.65	0.78

### 4.3 Missed Nursing Care (MISSCARE Part A)

**FIGURE 1:**
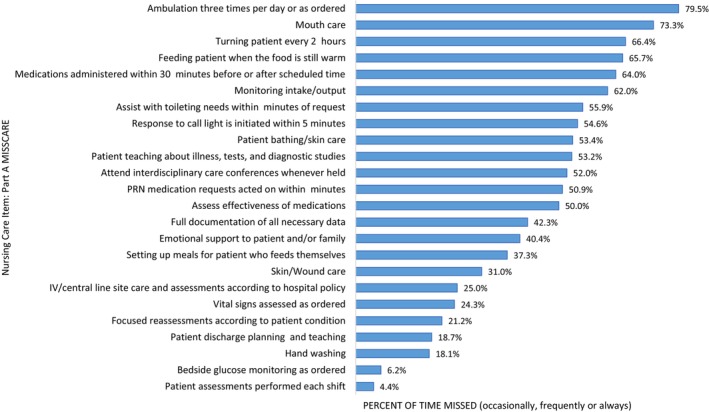
Frequency of missed nursing care (missed occasionally, frequently or always). [Colour figure can be viewed at wileyonlinelibrary.com]

The frequency at which the 24 nursing care items on Part A of the MISSCARE were missed is presented in Table 4. The items most frequently reported missed (reported occasionally, frequently or always missed) were ambulation (79.5%), mouth care (73.3%), turning every 2 h (66.4%) feeding patients while food is still warm (65.7%) and medications administered within 30 min before or after scheduled (64.0%). The items least frequently missed (reported as never or rarely missed) were patient assessments each shift (4.4%), bedside glucose monitoring as ordered (6.2%), hand washing (18.1%), discharge planning (18.7%) and vital signs as ordered (24.3%). Figure 1 displays the frequency at which items were reported as missed occasionally, frequently, or always.

**TABLE 4:** MISSCARE Part A: frequency of nursing care items missed (*n* = 161).

Variable		Never (0)	Rarely (1)	Occas. (2)	Freq. (3)	Always (4)
Rank		%	%	%	%	%
1	Ambulation three times per day as ordered	1.9	18.6	36.0	37.3	6.2
2	Mouth care	3.7	23.0	46.0	24.2	3.1
3	Turning patient every 2 h	3.7	29.8	41.6	24.2	0.6
4	Feeding patient when the food is still warm	6.9	27.5	48.8	16.9	0.0
5	Medications administered within 30 min before/after scheduled	8.7	27.3	42.9	20.5	0.6
6	Monitoring intake/output	8.1	30.0	38.8	21.9	1.3
7	Assist with toileting needs within 5 min of request	4.3	39.8	41.6	13.7	0.6
8	Response to call light is provided within 5 min	11.2	34.8	41.0	13.0	0.6
9	Bathing/skin care	9.3	37.3	44.1	9.3	0.0
10	Patient teaching	7.5	39.4	34.4	18.8	0.0
11	Attend interdisciplinary care conferences whenever held	10.8	37.3	26.6	22.2	3.2
12	PRN medication requests acted on within 15 min	9.3	39.8	41.0	9.9	0.0
13	Assess effectiveness of medications	3.8	46.3	41.9	8.1	0.0
14	Full documentation of all necessary data	13.0	44.7	34.8	7.5	0.0
15	Emotional support to patient and/or family	13.7	46.0	31.7	8.7	0.0
16	Setting up meals for patients who feed themselves	16.8	46.0	28.0	8.7	0.6
17	Skin/wound care	15.5	53.4	26.7	4.3	0.0
18	IV site care and assessment per hospital policy	26.3	48.8	21.9	3.1	0.0
19	Vital signs assessed as ordered	22.4	53.4	22.4	1.9	0.0
20	Focused reassessment according to patient status	38.1	40.6	18.1	3.1	0.0
21	Ensuring discharge planning	37.5	43.8	15.0	3.1	0.6
22	Hand washing	30.6	51.3	14.4	3.1	0.6
23	Beside glucose monitoring as ordered	46.6	47.2	6.2	0.0	0.0
24	Patient assessments done each shift	64.8	30.8	4.4	0.0	0.0

Abbreviations: Freq., frequently; Occas., occasionally.

### 4.4 Reasons for Missed Nursing Care (MISSCARE Part B)

The most frequently reported reasons for MNC (rated as a moderate or significant reason) were related to problems with labour resources; specifically, inadequate number of staff (78.8%), heavy admission and discharge activity (75.8%), inadequate number of assistive personnel (74.4%) and unexpected rise in patient volume and acuity (69.2%) (Table S1). The internal consistency of the MISSCARE Part B subscales was assessed using Cronbach's alpha, with reliability coefficients of 0.69 for labour resources, 0.71 for material resources, and 0.85 for communication/teamwork, indicating acceptable to strong internal consistency for these subscales.

### 4.5 Total Missed Care

A total missed care score was calculated for each participant by averaging scores (0–4) reported for each of the 24 items. The mean total missed care score for the sample (*n* = 161) was 1.4 (SD = 0.45), indicating that, on average, care was reported as missed between occasionally and rarely. The distribution of missed care scores was normal, supporting the use of parametric statistical analyses (Table 5).

**TABLE 5:** Structural empowerment, missed care, quality of care, patients last shift (*n* = 161).

Variable	Mean	SD
Total Structural Empowerment (CWEQ‐II total score)	22.8	3.38
Total missed nursing care	1.4	0.45
Quality of care	3.41	0.58
Number of patients cared for on last shift	5.1	1.23

### 4.6 Quality of Care

The mean quality of care score was 3.41 (SD = 0.58) on a scale ranging from 1 (poor) to 4 (excellent), indicating that participants perceived the quality of care on their units to be good to excellent (Table 5).

### 4.7 Relationships Between Structural Empowerment, Missed Care and Quality of Care

Spearman's rank correlation coefficients (*ρ*) were calculated to assess relationships between total CWEQ‐II (and its subscales) and missed care (Part A). Spearman's rank correlation was also used to assess relationships between missed care (Part A) and QOC, and between total CWEQ‐II (and subscales) and QOC. To control for multiple comparisons, adjusted *p*‐values were computed using the Benjamini–Hochberg method to limit the false discovery rate (Table 6).

**TABLE 6:** Spearman correlations: total CWEQ‐II score, subscales, total MNC and QOC (*n* = 161).

Variable		Spearman's rank correlation coefficient (*ρ*)							
	MNC	CWEQ‐II	Opp	Info	Support	Resource	Formal	Informal	QOC
MNC	—								
CWEQ‐II	−0.401**	—							
Opp	−0.104	0.491**	—						
Info	−0.246**	0.761**	0.233**	—					
Support	−0.300**	0.813**	0.334**	0.565**	—				
Resources	−0.482**	0.719**	0.211**	0.455**	0.473**	—			
Formal	−0.311**	0.770**	0.214**	0.489**	0.554**	0.530**	—		
Informal	−0.175^a^	0.677**	0.408**	0.452**	0.475**	0.304**	0.442**	—	
QOC	−0.432**	0.291**	0.167^a^	0.152	0.230**	0.282**	0.227**	0.193^a^	—

Abbreviations: CWEQ‐II, total CWEQ‐II score; Formal, formal power; Info, information subscale; Informal, informal power; MNC, missed nursing care; Opp, opportunity subscale; QOC, quality of care.

** *p* = 0.01 (2‐tailed).

^a^ Correlation no longer significant after adjustment.

A significant, moderate, inverse correlation was found between the total CWEQ‐II score and MNC (*ρ* = −0.401, *p <* 0.001), indicating that higher SE was associated with lower reported missed care. All empowerment subscales, except opportunity, were significantly and inversely correlated with MNC, suggesting that greater perceived access to information, support and resources was linked to less missed care. Among the subscales, access to resources had the strongest correlation with MNC (*ρ* = −0.482, *p* < 0.001). Formal power (*ρ* = −0.311, *p* = 0.002) and informal power (*ρ* = −0.175, *p* = 0.03) were also significantly associated with MNC; however, after adjusting for multiple comparisons, the correlation with informal power was no longer significant.

Missed care was significantly, moderately and inversely correlated with QOC (*ρ* = −0.432, *p* < 0.001) suggesting that nurses who missed more care perceived the quality of care on their unit to be poorer. SE (CWEQ‐II total score) was also correlated with QOC (*ρ* = −0.291, *p* = 0.001.) The subscale most strongly correlated with QOC was access to resources (*ρ* = −0.282, *p* = 0.002).

### 4.8 Exploratory Analyses

#### 4.8.1 Job, Role and Teamwork Satisfaction, Staffing Adequacy and Missed Care

Spearman's rank correlation coefficients (*ρ*) were calculated to assess relationships between job satisfaction, role satisfaction, satisfaction with teamwork, workload (patients cared for on last shift) and staffing adequacy (Table 7). *p*‐values were adjusted for multiple comparisons.

**TABLE 7:** Spearman's correlation (*ρ*) between MNC, staffing and job/role/teamwork satisfaction.

Variable	Spearman's rank correlation coefficient (*ρ*)					
	Total MNC	Staffing adequacy	Patients last shift	Job sat.	Role sat.	Team sat.
Total MNC	—					
Staffing adequacy	−0.251**	—				
Patients last shift	−0.011	−0.051	—			
Job sat.	−0.332**	0.324**	−0.040	—		
Role sat.	−0.164^a^	0.188^a^	−0.088	0.421**	—	
Team sat.	−0.233**	0.287**	−0.025	0.508**	0.342**	—

Abbreviations: Job sat., job satisfaction; role sat., role satisfaction; team sat., teamwork satisfaction.

** *p* = 0.01 (2‐tailed).

^a^ No longer significant after adjustment for multiple comparisons.

No significant correlation was found between the number of patients cared for on the last shift and MNC; however, significant weak, inverse correlations were found between nurses perceptions of staffing adequacy and MNC (*ρ* = −0.251, *p* = 0.0.001), nurses satisfaction with teamwork and MNC (*ρ* = −0.233, *p* = 0.003) and between nurses job satisfaction (*ρ* = −0.332, *p* < 0.001) and MNC.

#### 4.8.2 Geographical Region of US, Patients Cared for on Last Shift and Missed Care

A one‐way ANOVA was conducted to compare the number of patients cared for on the last shift across geographical regions of the US. There were no significant differences in the number of patients cared for on the last shift between geographical regions; however, a significant difference in MNC was found between regions, *F*(3, 157) = 6.06 *p* < 0.001. Post hoc comparisons using Tukey's HSD test revealed that nurses in the West reported significantly higher MNC (M = 1.61) compared to nurses in the South (M = 1.35) and nurses in the Northeast (M = 1.24) (Table 8).

**TABLE 8:** Missed nursing care by the geographical region of US.

Variable	Total MNC	
Geographical region of US (*N* = 161)	Mean	SD
Northeast (*N* = 45)	1.23	0.46
South (*N* = 35)	1.35	0.37
Midwest (*N* = 36)	1.38	0.41
West (*N* = 45)	1.61	0.45

#### 4.8.3 Magnet Status, Missed Nursing Care and Structural Empowerment

An independent samples *t*‐test was conducted to compare MNC between Magnet‐designated hospitals and non‐Magnet‐designated hospitals. The results indicated no significant difference in reported MNC between nurses working in Magnet vs. non‐Magnet hospitals (*p* = 0.832). This suggests that Magnet status alone was not associated with differences in MNC.

An independent samples *t*‐test was also performed to determine if there was a significant difference in overall SE (CWEQ‐II total score) between nurses from Magnet hospitals and non‐Magnet hospitals. No statistically significant difference in empowerment was found between Magnet and non‐Magnet nurses.

#### 4.8.4 Intention to Leave and Missed Nursing Care

A total of 25.5% of nurses in this study reported an intention to leave their positions. An independent samples *T*‐test was conducted to compare mean missed care scores between those who intended to leave and those who did not. Nurses who intended to leave their positions missed a significantly greater amount of care than nurses who did not intend to leave, *t*(159) = 4.645, *p* < 0.001.

#### 4.8.5 Regression Analysis for Predictors of Missed Nursing Care

Univariate and multivariate linear regression analyses were conducted to examine the association between SE variables and MNC, the primary outcome. A preliminary analysis assessed potential confounders, including hospital type, years of experience, age, gender and other demographic variables. As none showed a statistically significant relationship with MNC, they were excluded from the final model to avoid overfitting and to maintain model parsimony.

Additional workplace characteristics, such as job satisfaction, team satisfaction, staffing adequacy, Magnet status, patient load during the last shift and intention to leave, were also evaluated as predictors of MNC. Variables demonstrating significant associations in univariate analysis were included in the multivariate model, ensuring that the final model focused on the most relevant predictors and strengthened the robustness and applicability of the findings.

MNC was operationalised as the average frequency of missed care across multiple care items, with higher scores indicating more frequent missed care. Based on the Shapiro test, MNC was approximately normally distributed. Regression coefficients (*β*) quantified the strength and direction of associations, with 95% confidence intervals (CIs) used to assess precision. *p*‐values were reported for hypothesis testing (*β* = 0), with statistical significance defined as *p* < 0.05. Likert scale and ordinal variables were scaled numerically, meaning that each variable was treated as continuous for regression analysis. Regression coefficients (*β*) represent the expected change in the total missed care score for a one‐unit increase in the predictor variable, with each β coefficient representing the expected change in the total MNC score per one‐unit increase in the predictor.

In the final model, two factors emerged as significant predictors of MNC. Intention to leave significantly predicted MNC (*β* = 0.25, *p* = 0.002) and lower perceived access to resources predicted MNC (*β* = 0.18, *p* = < 0.001). Table 9 presents results of the univariate and multivariate regression analysis.

**TABLE 9:** Univariate and multiple regression for predictors of missed nursing care.

Variables	Univariate			Multivariate		
	Beta	95% CI	*p*	Beta	95% CI	*p*
Opportunity	−0.07	−0.19, 0.04	0.207	−0.02	−0.13, 0.10	0.747
Information	−0.14	−0.22, −0.06	< 0.001	−0.01	−0.11, 0.08	0.781
Support	−0.16	−0.24, −0.08	< 0.001	−0.02	−0.13, 0.08	0.648
Resources	−0.24	−0.31, −0.17	< 0.001	−0.18	−0.27, −0.09	< 0.001
Formal power	−0.18	−0.26, −0.10	< 0.001	−0.01	−0.11, 0.10	0.893
Informal power	−0.14	−0.24, −0.03	0.012	0.01	−0.10, 0.13	0.826
Magnet						
Yes	—	—		—	—	
No	−0.02	−0.19, 0.15	0.832	−0.01	−0.16, 0.14	0.910
Intend to leave						
Yes	—	—				
No	−0.35	−0.50, −0.20	< 0.001	−0.25	−0.41, −0.10	0.001
Patients last shift	−0.01	−0.07, 0.05	0.772	−0.04	−0.09, 0.02	0.164
Staffing adequacy (scaled)	−0.15	−0.24, −0.06	< 0.001	−0.04	−0.16, 0.07	0.424
Job satisfaction (scaled)	−0.22	−0.30, −0.13	< 0.001	0.00	−0.13, 0.13	0.975
Role satisfaction (scaled)	−0.12	−0.24, −0.01	0.034	0.05	−0.10, 0.20	0.518
Teamwork satisfaction (scaled)	−0.15	−0.23, −0.07	< 0.001	−0.10	−0.22, 0.01	0.087

Abbreviation: CI, confidence interval.

## 5 Discussion

This study examined the relationships between SE, MNC and nurse‐assessed quality of care in a national sample of 161 hospital‐based nurses from the US. No studies examining the relationship between SE and MNC could be found in the literature; therefore, this study contributes new knowledge to what is known about the relationship between the nurse work environment and MNC.

The primary aim of the study was to examine the relationship between SE of direct‐care, inpatient, medical–surgical nurses and MNC. Greater perceived SE (CWEQ‐II total score) was significantly associated with less missed care reported by nurses (*ρ* = −0.401, *p* < 0.001). A second aim of the study was to examine the relationship between MNC and nurse‐assessed quality of care. Results demonstrated that nurses who missed less care perceived the quality of care on their units to be higher (*ρ* = −0.429, *p* < 0.001).

Nurses in this sample reported high overall empowerment. The mean total CWEQ‐II was 22.8, which is somewhat higher than the moderate empowerment (CWEQ‐II total score of 16–20) that is often reported in other studies of clinical nurse empowerment (Albasal et al. 2022; Engström et al. 2025; İspir Demir et al. 2023). Perceived access to the specific empowerment structures of information, support and resources were significantly correlated with less missed care. The strongest correlation was between perceived access to resources and MNC (*ρ* = −0.482, *p* < 0.001). Laschinger (2012) defines resources as ‘one's ability to acquire the financial means, materials, time, and supplies required to do the work’ (p. 2). That access to resources was most strongly linked to lower missed care is consistent with many other studies of missed care, in which problems with labour resources and material resources are the most frequently reported reasons for missed care (Chiappinotto et al. 2022). Access to resources was one of two factors that emerged as significant predictors of MNC in the multivariate regression (the other was intention to leave), underscoring the importance of resource adequacy to the occurrence of missed care.

Kanter's theory (1993) asserts that ‘power’ is required to access the workplace empowerment structures. Two types of power are described: formal and informal power. Formal power derives from job activities and their perceived importance to organisational goals, employee visibility, opportunity for creative work and autonomy in decision‐making. Informal power is derived from relationships and alliances at all levels of the organisation (Laschinger 2012). Both types of power facilitate access to empowerment structures. In this study, nurses informal power was higher than their formal power, however informal power was not associated with MNC. Formal power, on the other hand was associated with missed care: as formal power increased, missed care decreased (*ρ* = −0.311, *p* < 0.001). These findings suggest that improving clinical nurses' visibility in hospitals, promoting recognition of the importance of nurses' work in hospitals and increasing nurses' autonomy in decision‐making may contribute to reducing MNC.

### 5.1 Characteristics of the Study Sample

The characteristics of the nurses in this study differed in several ways from those of the general population of nurses in the US at the time. 72.7% of the study sample had a BSN degree, compared with 44.6% of nurses nationally at the time; 52.5% were under the age of 35, compared to 19.2% nationally and 39.3% were represented by a labour union, compared to 17.5% nationally (U.S. Department of Health and Human Services 2019). Most (77.6%) nurses were from Magnet hospitals; therefore, Magnet facilities were over‐represented, considering that at the time, only 9.5% of hospitals in the US had Magnet designation (*Number of Hospitals in the United States with Magnet Status* 2020).

In addition to these attributes, nurses in the study reported high job satisfaction (85.1% were either satisfied or very satisfied with their positions), high role satisfaction (95.7% were satisfied or very satisfied with being a nurse) and high satisfaction with teamwork (83.8% were satisfied or very satisfied with teamwork). They also had a favourable workload. The average number of patients cared for per shift by nurses in this study was 5.1, compared to 6.3 patients per shift in New York State (Lasater et al. 2021) and 5.4 in Illinois (Lasater Aiken et al. 2020).

It is therefore noteworthy that these nurses, who were highly empowered, predominantly BSN educated, had high job, role and teamwork satisfaction, enjoyed a favourable workload and were mostly from Magnet hospitals, still missed a substantial amount of nursing care. More than half of the 24 items on the MISSCARE survey were reported missed occasionally, frequently or always, and some items such as ambulation, turning, mouth care, feeding patients and administering medications on time were reported missed 64%–79.5% of the time. The amount of care missed by the nurses in this study is generally consistent with the frequency and patterns of missed care reported in many other studies (Chaboyer et al. 2021; Kalisch and Xie 2014). Of note, despite high job, role and teamwork satisfaction, 25.5% of the sample still reported intention to leave. The findings suggest that other factors, which are not yet identified, are involved in precipitating missed care, and underscore the complexity of the problem.

### 5.2 Correlates of Missed Nursing Care

No individual nurse characteristics were significantly associated with MNC, consistent with previous studies (Chiappinotto et al. 2022). The only demographic factor related to missed care was the geographic region of the country. Nurses from the Western region of the USA reported significantly more missed care (M = 1.61) than nurses from the Northeast (M = 1.23) and the South (M = 1.35), even though there was no significant difference in the number of patients cared for on the last shift between regions. Data on factors that might explain these differences, such as regional differences in staffing models, differences in care delivery models, local hospital policies, or other factors that varied by region, were not collected in this study. Exploring these types of factors and their relationship to MNC represents an opportunity for future research.

Magnet status was not related to MNC in this study. No differences were found in the amount of MNC reported by nurses from Magnet vs. non‐Magnet hospitals. This is in contrast to the findings of Kalisch and Lee (2014) who did identify a relationship between Magnet status and MNC; however, few studies have examined this relationship, and results are conflicting. For example, Tubbs‐Cooley et al. (2017) did not find a relationship between Magnet status and MNC in a study of missed care in neonatal intensive care units. Additional research is needed to clarify the relationship between Magnet status and MNC.

Although Magnet status is reported as being associated with improved patient outcomes, in this study, there was no difference in nurse‐assessed quality of care between Magnet and non‐Magnet hospitals. Technically, missed care is a process measure, not an outcome measure, and research on the Magnet recognition programme has focused primarily on outcomes of care; however, additional research on Magnet status, care processes and MNC could prove interesting.

Problems with labour resources (such as insufficient staff, poor use of available staffing resources and inadequate staff to meet episodic increases in patient care demands) are the most frequently identified reasons for MNC (Gong et al. 2025). In the present study, nurses also reported inadequate staffing most frequently as a reason for MNC (78.8% of the time); however, when the relationship between workload, i.e., the number of patients cared for per nurse, was examined, no significant relationship was found. Interestingly, a significant relationship was identified between nurses *perceptions* of staffing adequacy and MNC, indicating that nurses who felt that their units were better staffed reported significantly less MNC (*ρ* = −0.251, *p* < 0.001). Paradoxically, when the relationship between the actual number of patients cared for on the last shift and perceptions of staffing adequacy was examined, there was no significant relationship. This suggests that subjective factors, such as perceptions and feelings related to the work environment, may influence MNC more than currently appreciated, and it also suggests that factors other than the number of patients cared for may influence nurses perceptions of their workload.

Other subjective factors, such as job satisfaction, satisfaction with teamwork, and intention to leave were significantly associated with MNC. Higher job satisfaction was significantly associated with less missed care (*ρ* = −332, *p* < 0.001), as was higher satisfaction with teamwork (*ρ* = −0.233, *p* < 0.001). Nurses who intended to leave their positions reported significantly greater MNC, *t*(159) = 4.59, *p* < 0.001. It is noteworthy that 25.5% of the sample reported intending to leave their positions, despite high job satisfaction, high empowerment, a favourable workload and most working in Magnet facilities. This is slightly higher than the 21% intention to leave nationally at the time (Koehler and Olds 2022). Intention to leave emerged as one of two predictors of missed care (the other was access to resources), underscoring its importance to missed care. This suggests that proactively assessing intention to leave and enhancing factors known to promote retention may potentially contribute to reducing missed care.

According to the Missed Care Model (Kalisch and Xie 2014), MNC occurs when environmental antecedents interact with the nursing process and nurses' internal processes to shape decisions about what care to deliver and what to miss when patient care demands exceed available resources. Internal processes include (1) team norms; (2) decision‐making processes; (3) internal values and beliefs; and (4) habits (Kalisch and Xie 2014). Studies have identified numerous environmental antecedents but few studies have examined *how* nurses make decisions about what care to omit and what to deliver; that is, how nurses internal processes may contribute to decision‐making. It is possible that subjective factors, such as interpersonal interactions and events in the workplace, and feelings arising from these factors, represent subjective, internal factors that are contributing to MNC. A previous study by Tubbs‐Cooley et al. (2019) found that nurses subjective perceptions of workload were more strongly associated with MNC than their objective workload. A recent study by Bayadsi et al. (2025) examined how nurses subjective perceptions of workload may contribute to MNC, finding that subjective perceptions of workload significantly moderated the relationship between nurses' objective workload and MNC. Additional research is needed to examine and clarify how internal, subjective factors may interact with other antecedents to influence MNC. Since few interventions have been developed or implemented, examining the contribution of subjective factors to MNC may open up new avenues for developing interventions to reduce missed care.

### 5.3 Limitations

This study has several limitations. Some characteristics of the participants, such as their younger age, higher educational preparation, greater union representation and high percentage from Magnet hospitals differ from the characteristics of the general population nurses in the US, and these differences may limit the generalisability of the results to other groups of nurses. Recruiting participants from attendees of the ANCC National Magnet conference may also have introduced sampling bias, as only a very small percentage of nurses in the US attend Magnet conferences. Results should be considered in context of these limitations.

SE is a core characteristic of the Magnet work environment. Because most of the nurses in the sample (77.6%) were from Magnet hospitals, and because the data was collected from both Magnet and non‐Magnet nurses attending a Magnet conference, it is likely that empowerment in this sample was higher than would be found in other nurses and settings. Non‐Magnet nurses who were able to attend the conference may have been more highly empowered than other nurses in their hospitals. As noted above, the overall empowerment score of the sample was 22.8, which is higher than the more moderate empowerment (scores of 16–20) often found in studies of nurse empowerment.

However, it is important to note that in this study, it was empowerment that was significantly linked to MNC, and not Magnet status. There was no difference in the amounts of missed care reported by nurses from Magnet and non‐Magnet hospitals, and furthermore, there was no difference in overall empowerment between Magnet and non‐Magnet nurses. It is therefore reasonable to speculate that nurses' SE may be linked to MNC, at whatever level empowerment exists in a setting, whether it is a Magnet or non‐Magnet environment. Additional research is needed to clarify the relationship between empowerment and missed care in nurses across a wider spectrum of empowerment.

Since all the data collected in this study, including data on the number of patients cared for on the last shift, were collected through self‐report, there is a risk of recall bias. Since nurses were reporting on their own performance, there is a risk of response bias, particularly social desirability bias, which should be considered when interpreting the findings. Finally, because the study design was non‐experimental and correlational, the ability to draw conclusions about causal relationships between variables is limited.

### 5.4 Implications for Practice

This study identifies SE as a work environment factor which is significantly associated with MNC, suggesting that increasing empowerment may contribute to reducing MNC. In order to increase nurses' empowerment, first it must be assessed, and currently, the empowerment of clinical nurses is not routinely assessed on medical–surgical units. Leaders and managers should consider periodically monitoring nurse empowerment at the unit level and taking targeted and meaningful steps at the unit level to increase and sustain empowerment as one strategy to reduce missed care. Specifically, leaders should consider strategies to bolster nurses formal power, such as enhancing their visibility, highlighting the importance of nursing practice to hospital outcomes, supporting opportunities for creative work, and increasing flexibility and autonomy in decision‐making.

Intention to leave and perceived access to resources emerged as significant predictors of missed care; therefore, leaders should pay close attention to unit resource levels and respond to problems rapidly and visibly in order to support nursing care delivery. Periodic monitoring of nurses job satisfaction and other factors related to intention to leave may provide an opportunity to intervene and reduce levels of missed care, while at the same time retaining staff.

MNC remains a serious, ongoing problem for patients and nurses. Although well‐studied, it continues to occur at levels similar to when first described. Solutions are needed. Because the problem is so complex, it is likely that multiple interventions, each of which will incrementally reduce the occurrence of missed care, will be needed. This study suggests that important antecedents remain unidentified, and that nurses subjective perceptions of work environment factors, such as their perceptions of staffing adequacy, may be contributing to missed care more than currently understood. More research is needed on how subjective, perceptual factors may interact with other antecedents of missed care to precipitate missed care. Nurse researchers should consider expanding their investigation of causes to factors not previously evaluated, and to exploring how nurses internal factors influence decision‐making about care delivery. Finally, nurse leaders in clinical settings should actively and visibly support research on missed care in clinical settings; to generate new knowledge on missed care, to monitor the effectiveness of nursing practice (VanFosson et al. 2016) and to generate evidence that can link nursing practice to the quality outcomes which drive reimbursement, thereby contributing to our understanding of the economic value of nursing practice.

## Conflicts of Interest

The authors declare no conflicts of interest.

## Supporting information


**File S1.** STROBE Statement—Checklist of items that should be included in reports of *cross‐sectional studies*.

**Figure S1.** Study flow chart.

**Table S1.** Reasons for missed nursing care: MISSCARE Part B (*n* = 161).

## Data Availability

The data that support the findings of this study are openly available in Open Science Framework at https://osf.io/zvxyn/, reference number https://doi.org/10.17605/OSF.IO/ZVXYN.
