# Supplementary material for: Examining the Relationship Between Hospital Nurses' Structural Empowerment, Missed Nursing Care and Quality of Care: A Cross‐Sectional Study
Source: J Clin Nurs. 2025 May 23;35(1):194–207. doi: 10.1111/jocn.17816 (PMC12667010; doi:10.1111/jocn.17816)
Supplement: Supplementary file 2 — Figure S1. Study flow chart. [file JOCN-35-194-s001.docx]

**Supplemental Figure 1**

*Study Flow Chart*

Enrolled and completed survey

N=185

Eligible surveys included

N=161

Surveys assessed for eligibility

N=185

Excluded (total =24)

INELIGIBLE ROLE = 16

- (i.e. management, educator, not direct care nurse, charge without patients, advanced clinician)

INELIGIBLE SETTING = 8

- (not working on medical surgical unit of acute care hospital)
