# Supplementary material for: Examining the Relationship Between Hospital Nurses' Structural Empowerment, Missed Nursing Care and Quality of Care: A Cross‐Sectional Study
Source: J Clin Nurs. 2025 May 23;35(1):194–207. doi: 10.1111/jocn.17816 (PMC12667010; doi:10.1111/jocn.17816)
Supplement: Supplementary file 3 — Table S1. Reasons for missed nursing care: MISSCARE Part B (n = 161). [file JOCN-35-194-s003.docx]

**Supplemental Table 1**

*Reasons for Missed Nursing Care: MISSCARE Part B (n=161)*

| Variable | | Not a reason | Minor reason | Moderate reason | Significant Reason | Moderate and Significant Reason |
| --- | --- | --- | --- | --- | --- | --- |
|  |  | % | % | % | % | % |
| Labor Resources | |  |  |  |  |  |
|  | Inadequate number of staff | 2.50 | 18.80 | 36.90 | 41.90 | 78.80 |
|  | Unexpected rise in patient volume and/or acuity on unit | 3.80 | 27.00 | 40.90 | 28.30 | 69.20 |
|  | Heavy admission/discharge activity | 4.30 | 19.90 | 39.80 | 36.00 | 75.80 |
|  | Inadequate number of assistive personnel | 5.10 | 20.30 | 36.10 | 38.60 | 74.70 |
|  | Urgent patient situations | 5.60 | 30.40 | 42.20 | 21.70 | 63.90 |
|  |  |  |  |  |  |  |
| Material Resources | |  |  |  |  |  |
|  | Medications were not available when needed | 5.60 | 36.00 | 33.50 | 24.80 | 58.30 |
|  | Supplies/equipment not available when needed | 8.10 | 47.80 | 33.50 | 10.60 | 44.10 |
|  | Equipment not functioning properly when needed | 13.20 | 49.10 | 28.90 | 8.80 | 37.70 |
| Variable | | Not a reason | Minor reason | Moderate reason | Significant Reason | Moderate and Significant Reason |
|  |  | % | % | % | % | % |
| Communication | |  |  |  |  |  |
|  | Unbalanced patient assignments | 8.20 | 40.30 | 35.80 | 15.70 | 51.50 |
|  | Assistant did not communicate that care was not done | 8.70 | 41.00 | 35.40 | 14.90 | 50.30 |
|  | Inadequate hand-off from previous shift or sending unit | 18.60 | 54.00 | 21.10 | 6.20 | 27.30 |
|  | Communication breakdowns with medical staff | 19.40 | 50.60 | 21.30 | 8.80 | 30.10 |
|  | Communication breakdowns with support departments | 20.80 | 50.30 | 24.50 | 4.40 | 28.90 |
|  | Lack of back-up support from team members | 21.10 | 43.50 | 26.70 | 8.70 | 35.40 |
|  | Other departments did not provide the care needed | 21.70 | 49.10 | 23.00 | 6.20 | 29.20 |
|  | Communication breakdowns within the nursing team | 24.20 | 53.40 | 16.10 | 6.20 | 22.30 |
|  | Caregiver off unit or unavailable | 31.10 | 49.10 | 15.50 | 4.30 | 19.80 |
